# Safety and Preliminary Efficacy of a Novel Host-Modulatory Therapy for Reducing Gingival Inflammation

**DOI:** 10.3389/fimmu.2021.704163

**Published:** 2021-09-13

**Authors:** Hatice Hasturk, Fabian Schulte, Melissa Martins, Homa Sherzai, Constantinos Floros, MaryAnn Cugini, Chung-Jung Chiu, Markus Hardt, Thomas Van Dyke

**Affiliations:** ^1^Center for Clinical and Translational Research, The Forsyth Institute, Cambridge, MA, United States; ^2^Department of Applied Oral Sciences, The Forsyth Institute, Cambridge, MA, United States; ^3^Center for Salivary Diagnostics, The Forsyth Institute, Cambridge, MA, United States; ^4^Department of Developmental Biology, Harvard School of Dental Medicine, Boston, MA, United States; ^5^Epidemiology and Biostatistics, The Forsyth Institute, Cambridge, MA, United States; ^6^Department of Oral Medicine, Infection and Immunity, Harvard School of Dental Medicine, Boston, MA, United States

**Keywords:** inflammation, lipid mediators, resolution, gingivitis, periodontal inflammation, host modulation

## Abstract

**Background:**

Periodontal disease is among the sixth most common inflammatory diseases worldwide with high risk to promote complications from other inflammatory diseases including diabetes, cardiovascular disease and Alzheimer’s Disease. Failure of active resolution of inflammation pathways is implicated in pathogenesis of periodontal diseases, including gingivitis. Lipoxin A4 (LXA4), a member of the specialized pro-resolving lipid mediators (SPMs) that drive resolution of inflammation *via* GPC-receptor mediated pathways, offered therapeutic advantages in preclinical models of periodontitis.

**Methods:**

We conducted a randomized, placebo-controlled, parallel-group Phase 1 clinical trial to determine the safety and preliminary efficacy of an LXA4 analog in patients with gingival inflammation. One hundred twenty-seven (127) individuals were randomized to daily use of an oral rinse containing a LXA4 mimetic, methyl ester-benzo-lipoxin A4 (BLXA4), placebo rinse or a no-rinse control group for 28 days. Treatment emergent adverse events (TEAEs) were assessed for safety, the primary outcome. Secondary outcomes included the change in the level of gingival inflammation and periodontal pocket depth (PD). Serum SPMs were monitored using targeted lipid mediator lipidomics to assess potential systemic impact of BLXA4.

**Results:**

The frequency of TEAEs was similar in BLXA4 and placebo-treated groups with no study-related SAEs. Once-daily rinsing with BLXA4 for 28-days resulted in a greater decrease in gingival inflammation compared to placebo rinse and no-rinse control groups (mean change: 0.26 GI unit *vs* 0.21 and 0.17, respectively). PD reduction was also greater with BLXA4 oral rinse compared to placebo and no-rinse groups (mean reduction: 1.23 mm *vs*. 0.71 mm and 0.46 mm, respectively). Topical application of BLXA4 increased serum levels of SPMs.

**Conclusion:**

Treatment with BLXA4 reduces local inflammation, and increases abundance of pro-resolution molecules systemically, which may dampen inflammation that can mediate progression and course of inflammatory diseases beyond periodontitis.

**Clinical Trial Registration:**

ClinicalTrials.gov, identifier (NCT02342691).

## Introduction

Inflammation is the normal host tissue response to infection and injury. However, uncontrolled and unresolved inflammation contributes to a range of chronic human conditions such as arthritis, cardiovascular diseases and periodontitis (1–3). Periodontitis, with an overall prevalence of 45%–50% in the population, is the sixth most common human disease (4, 5). Periodontitis is a progressive disease in which microbial etiologic factors induce an inflammatory cascade that leads to destruction of the organ supporting the teeth, the periodontium (6–9). As with all inflammatory diseases, the arachidonate-derived eicosanoids play a key role in the initiation and pathogenesis of periodontitis (10–12). The associated gram negative bacteria of the dysbiotic biofilm on and around the teeth, such as *Porphyromonas gingivalis* and *Tannerella Forsythia*, initiate an influx of neutrophils and neutrophil cyclooxygenase-2 activation leading to increased lipid mediators such as prostaglandin E_2_ *in situ* (13). Prostaglandin E_2_ is a potent activator of osteoclast-mediated bone resorption, the hallmark of periodontal disease (12, 14, 15). Other lipid mediators such as leukotriene B_4,_ produced mainly by activated leukocytes, initiate accumulation and superoxide generation by neutrophils within inflamed sites stimulating the release of granule-associated enzymes and bone resorption (16–18). Together with other eicosanoids, PGE_2_ mediates inflammation and periodontal tissue destruction (19, 20).

It is now widely recognized that endogenous pro-resolving lipid mediators actively participate in regulating host responses and orchestrate resolution of inflammation (21, 22). Lipoxins, the product of lipoxygenase- lipoxygenase interactions, are trihydroxy products of arachidonic acid that actively drive resolution of inflammation; they are typified by lipoxin A_4_ (LXA_4_). In addition, the previously unappreciated role of aspirin-triggered transformation circuits in producing the endogenous anti-inflammatory 15R-epimers of LXA_4_ has led to a better understanding of pro-resolution signaling networks, including a series of complex cellular and chemical reactions and tissue trafficking events (23). For example, lipoxins not only reduce the influx of neutrophils, but also stimulate the non-phlogistic uptake of apoptotic neutrophils by tissue-resident macrophages, a prerequisite for resolution of inflammation (24, 25).

Current therapies for gingivitis and periodontitis remain inadequate. We demonstrated in animal models of periodontitis that SPMs, including lipoxins and resolvins (i.e., LXA_4_, RvE1), are capable of preventing and treating periodontal disease by resolving local inflammation allowing soft and bone tissues to regenerate, as well as damping systemic inflammation induced by the local disease and rebiosis of the oral microbiome (26–29).

This Phase 1 randomized clinical trial was designed to determine in humans the impact of a stable analog mimetic of lipoxin A_4_ denoted BLXA_4_ in inflammatory periodontal disease. In comparison with native LXA_4_, the methyl-ester of BLXA_4_ was found to be stable in an *in-vitro* stability assay system containing eicosanoid oxido-reductase, and this particular analog was most effective at inhibiting polymorphonuclear neutrophil infiltration in a murine peritonitis model (approximately 32% reduction). In the same model system, the benchmark compound, aspirin-trigger lipoxin A_4_ (ATLa), resulted in 40% reduction of polymorphonuclear neutrophil infiltration (30).

SPMs in their pure form are low molecular weight hydrophobic molecules that easily penetrate mucosa making them ideal for topical application. These physical properties also suggest that topical SPMs will be readily absorbed into the circulation and have systemic impact. Thus, an important question also addressed herein is the inflammatory mediator profile in serum after oral topical administration of BLXA_4_; not just BLXA_4_ levels in serum but the inflammatory lipid mediator lipidome in total.

Based on these *in vitro* and preclinical *in vivo* observations, we aimed to assess the safety and preliminary efficacy of the use of proresolving agent, BLXA_4_, in a mouthwash formulation in patients with gingival inflammation.

## Materials and Methods

### Ethical Considerations and Institutional Review Board Approvals

The protocol, screening and study consent forms, recruitment materials, and all subject materials were reviewed and approved by the Forsyth Institute IRB before any subject was enrolled (IRB approval no. 15-04). All amendments to the protocol were reviewed and approved by the IRB before the changes were implemented in the study. The study was registered at clinicaltrials.gov (NCT03196618).

#### Ethical Conduct of the Study

This study was conducted in full conformity with the principles set forth in The Belmont Report: Ethical Principles and Guidelines for the Protection of Human Subjects of Research, as drafted by the United States National Commission for the Protection of Human Subjects of Biomedical and Behavioral Research (April 18, 1979) and codified in 45 Code of Federal Regulations (CFR) Part 46 and/or the International Council for Harmonization (ICH) Guidelines for Good Clinical Practice E6. The study was conducted according to the principles expressed in the Declaration of Helsinki and International Committee of Medical Journal Editors’ policy was followed.

#### Subject Information and Consent

Informed consent process was initiated prior to the individual agreeing to participate in study screening at the Screening Visit or to participate in the study at the Enrollment/Baseline Visit 1, and continued throughout study participation. An extensive discussion of risks and possible benefits of study participation was provided to interested individuals and informed consent was obtained from all participants prior to any study-related assessments or procedures.

### Study Design and Randomization

This phase 1/2 study comprised 3 groups in a randomized, placebo-controlled, double-blind parallel group design. Qualified participants were enrolled in the study and received baseline evaluations within 7-10 days of screening. Participants were randomized into treatment groups using a permuted block randomization schedule. The randomization sequence was generated by the study biostatistician with a random number generator. The randomization scheme was kept in the sole possession of the unblinded clinician who was also responsible for providing study treatments with instructions to participants and assessing the protocol and product compliance throughout the study.

### Participant Selection, Enrollment, and Compliance Assessment

Participants were recruited from the volunteer pool at the Forsyth Institute Center for Clinical and Translational Research (CCTR). Subjects were healthy adults, aged 18 through 65 years, with gingivitis/periodontitis presenting existing gingival inflammation as defined by MGI ≥ 2.0. The treatment group (the methyl-ester of BLXA4 at 1μM concentration in an oral rinse) and the placebo rinse group (same oral rinse formulation without BLXA4) consisted of 50 subjects each while the no-rinse control group consisted of 27 subjects in a 2:2:1 randomization scheme. These group sizes were planned to allow for a 20% dropout rate. Subjects in all groups were instructed to maintain their regular brushing habits and use the assigned mouth rinse once daily after morning toothbrushing for 28 days.

Those with current medical conditions or on medications known to affect periodontal tissues or interfere with any of the study outcomes were excluded. In addition, individuals with orthodontic appliances, pregnant and nursing women, and current or former smokers within 1 year of enrollment were excluded due to potential confounding effects on study outcomes.

After baseline, participants were evaluated on Day 3 (only for safety and compliance) and at days 7, 14, 21 and 28 for safety, compliance and efficacy measurements. A follow up phone call at Day 90 was also performed for safety evaluation. Compliance of study rinse use was assessed at all follow-up study visits by measurement of the remaining volume and review of participant logs. Expected volume returned was calculated as the volume dispensed - (number of days since last study visit x 30 ml). A participant was considered to have “overused” the rinse if used more than (>120%) prescribed use. A participant was considered to have “underused” the rinse if less than (<80%) prescribed use. In addition, participants were exclusively interviewed by a separate clinician for oral hygiene practices, concomitant medication use, and adverse events; deviations were recorded, and participants were reinstructed, if needed.

### Outcome Measures

Safety endpoint (primary) was evaluated by adverse event (AE) assessment, change in vital signs, clinical laboratory tests (blood chemistry, complete blood count and urinalysis), and oral examinations for mucosal irritancy and inflammation. In addition, supragingival plaque was analyzed for microbial species to evaluate shifts in the dental biofilm using DNA-DNA hybridization checkerboard technique (31) (**Supplementary Material-Materials and Methods** for details). Secondary efficacy endpoints included MGI and BOP as the indices of gingival inflammation; PD and CAL were measured as exploratory efficacy endpoints (32) Absorption of BLXA_4_ through the oral mucosa and profiling of specialized lipid mediators in serum before and after treatment on Day 28 were analyzed using LC-MS/MS lipidomics at the Forsyth Institute Center for Salivary Diagnostics Mass Spectrometry Core using the method of Colas et al. (31).

### Examiner Calibration

Two trained and calibrated examiners (HS and MM) blinded to study treatments were responsible for all clinical oral measurements and safety assessments in a given subject for the course of the study. An intra- and inter-examiner calibration exercise was performed as in (33) with a minimum κ coefficient of 0.8 for intra-examiner and of 0.7 for inter-examiner alignment.

### Study Products

The study product, BLXA_4_ containing oral rinse, received an Investigational New Drug designation (IND #: 107061; Sponsor: Van Dyke) from the FDA prior to study initiation. The topical oral rinse dosage form of BLXA_4_ consisted of drug substance prepared at a concentration of 1.0 μM in an aqueous vehicle solution containing the inactive components shown in **Supplementary Table 1**. BLXA_4_ is a member of a new class of chemically and metabolically stable lipoxin analogs featuring a replacement of the tetraene unit of native LXA_4_ with a substituted benzo-fused ring system (30). The full chemical name of the BLXA_4_ drug substance is (5S, 6R, E)-methyl 5,6-dihydroxy-8-(2-((R,E)-3-hydroxyoct-1-enyl) phenyl) oct-7-enoate (9, 12-benzo LXA_4_). The placebo preparation consisted of formulated oral rinse without BLXA_4_ and was identical to the test rinse in color, appearance, and taste. Both the BLXA_4_ oral rinse and placebo oral rinse were prepared and packaged in amber high-density polyethylene bottles by a Good Manufacturing Practice facility (Avanti Polar Lipids, Inc., Alabaster, AL) as a contract synthesis. Rinses were stored at 2-8°C until dispensing to participants and were used at room temperature within 7-10 days. Additional information on products used in this study is provided in **Supplementary Material**.

### Preclinical Safety and Toxicology Studies

As part of the NIH grant supporting the randomized clinical trial proposal and as required for the FDA IND submission, nonclinical pharmacodynamics studies were performed with BLXA_4_ to address the primary mechanism of action and safety prior to human clinical testing at a commercial Good Laboratory Practices compliant laboratory (Calvert Laboratories, Inc., Scott Township, PA). Nonclinical safety pharmacology studies performed included an *in vitro* study evaluating the effect of BLXA_4_ on the hERG potassium channel, and *in vivo* studies of the effect of BLXA_4_ on central nervous system, pulmonary, and cardiovascular function in rats and dogs. The pharmacokinetic parameters and toxicokinetic exposure to BLXA_4_ were also examined in both rats and dogs. Furthermore, a 28-day oral irritancy study was performed in male and female rats to assess the potential of BLXA_4_ oral rinse to cause mucosal irritancy and to assess its effects on the healing of abraded oral mucosa. The details of the preclinical testing and results of these studies are provided in **Supplementary Material** and **Supplementary Tables 2–4**.

### Targeted LC-MS/MS-Based Metabololipidomics

Peripheral blood serum samples were collected at baseline and 28 days *via* venipuncture, centrifuged at 2300 rpm and frozen at -80°C until analysis (**Supplementary Material** and **Supplementary Figure 1**). LC-MS/MS-based metabololipidomics profiling was performed as previously described (34, 35). Briefly, on the day of analysis, serum samples were thawed and methanol (9 volumes, -20°C, 60 min) containing 500 pg of deuterated internal standards d4-LTB_4_, d4-PGE_2_, d5-LXA_4_, d5-MaR2, d5-RvD2, and d8-5S-HETE were added to precipitate proteins and facilitate quantification of sample recovery. Lipid mediators were extracted using Isolute C18-silica reverse-phase cartridges (Biotage, Uppsala, Sweden). Samples were eluted with 6 ml methylformate and taken to dryness using Speedvac or nitrogen stream and suspended in 60 µl methanol/water (50:50 v/v) for LC-MS/MS from which 40 µl were injected into mass spectrometer. The LC-MS/MS system consisted of a Sciex 6500 QTrap equipped with a Shimadzu Nexera XL HPLC. An Agilent Poroshell 120 EC-C18 column (100 mm × 4.6 mm × 2.7 μm) was used for separation with a gradient of methanol/water/acetic acid of 50:50:0.01 (v/v/v) to 98:2:0.01 at a 0.5-ml/min flow rate and a column temperature of 50°C. To monitor and quantify the levels of the targeted LMs, we developed a multiple-reaction-monitoring (MRM) method with signature ion fragments for each molecule based on published criteria (36, 37) with at least six diagnostic ions. Calibration curves were obtained using both synthetic and authentic LM mixtures; these included d4-LTB_4_, d4-PGE_2_, d5-LXA_4_, d5-MaR2, d5-RvD2, and d8-5S-HETE, LXA_4_, LXB_4_, LTB4, PGE_2_, PGD_2_, RvE1, RvD1, RvD2, RvD3, RvD4, RvD5, PD1, MaR1 and BLXA_4_ at 0.1, 1, 10, and 100pg (**Table 1**). Linear calibration curves for each analyte were obtained with r^2^ values in the range of 0.98–0.99. Quantification was carried out based on peak area of the MRM transition and the linear calibration curve for each compound using the Multiquant 3.0.1 software (SCIEX, Framingham, MA, USA).

**Table 1:** MRM-transitions for the detection of SPMs.

Compound	RT [min]	Precursor mass [m/z]	Product ions [m/z]	DP [V]	CE [V]
d4-LTB4 (339.3/197.2)	14.2	339.3	197.2	-80	-22
d4-PGE2 (355.3/193.2)	11.0	355.3	193.2	-80	-25
d5-LXA4 (356.3/115.2)	11.7	356.3	115.2	-80	-19
d5-MaR2 (364.3/221.2)	14.6	364.3	221.2	-80	-28
d5-RvD2 (380.3/141.2)	11.0	380.3	141.2	-80	-25
d8-5S-HETE (327.2/116.1)	17.7	327.2	116.1	-80	-17
9,12-Benzo LXA4	17.4	449.3	357.3	-80	-13
9,12-Benzo LXA4	17.4	375.2	159.1	-80	-13
14-HDHA (343.2/205.1)	17.5	343.2	205.1	-80	-17
15-HETE (319.1/218.9)	28.5	319.1	218.9	-80	-20
15-HETE (319.1/256.9)	28.5	319.1	256.9	-80	-20
15-HETE (319.1/301.0)	28.5	319.1	301.0	-80	-20
17-HDHA (343.2/245.1)	17.5	343.2	245.1	-80	-17
18-HEPE (317.2/259.1)	16.3	317.2	259.1	-80	-16
LTB4 (335.2/195.1)	14.2	335.2	195.1	-80	-22
LXA4 (351.2/115.1)	11.7	351.2	115.1	-80	-20
LXA4 (351.2/235.1)	11.7	351.2	235.1	-80	-20
LXB4 (351.2/221)	11.0	351.2	221	-80	-20
MaR1 (359.2/221.1)	13.9	359.2	221.1	-80	-20
MaR1 (359.2/250.1)	13.9	359.2	250.1	-80	-20
MaR2 (359.2/221.2)	14.6	359.2	221.2	-80	-28
PD1 (359.2/153.1)	13.8	359.2	153.1	-80	-21
PD1 (359.2/181.1)	13.8	359.2	181.1	-80	-19
PGD2 (351.3/233.1)	11.1	351.3	233.1	-80	-16
PGE2 (351.3/175.1)	11.0	351.3	175.1	-80	-25
PGE2 (351.3/189.1)	11.0	351.3	189.1	-80	-25
RvD1 (375.2/121.1)	11.6	375.2	121.1	-80	-40
RvD1 (375.2/215.1)	11.6	375.2	215.1	-80	-26
RvD2 (375.2/141.1)	11.1	375.2	141.1	-80	-21
RvD2 (375.2/175.1)	11.1	375.2	175.1	-80	-30
RvD3 (375.2/147.1)	11.2	375.2	147.1	-80	-25
RvD3 (375.2/181.1)	11.2	375.2	181.1	-80	-22
RvD4 (375.2/101.1)	12.6	375.2	101.1	-80	-22
RvD4 (375.2/255.1)	12.6	375.2	255.1	-80	-25
RvD5 (359.2/199.1)	13.7	359.2	199.1	-80	-21
RvD5 (359.2/261.1)	13.7	359.2	261.1	-80	-20
RvE1 (349.2/161.1)	8.8	349.2	161.1	-80	-25
RvE1 (349.2/195.1)	8.8	349.2	195.1	-80	-22

### Data Analyses and Statistical Methods

#### Analysis Population

The safety population consisted of all subjects who were randomized into a study arm and completed baseline visit. The efficacy population consisted of all subjects from the safety population who had a baseline and at least 1 post-baseline assessment of 1 or more of the secondary efficacy outcome measures.

#### Sample Size Determination and Power

For the BLXA_4_ oral rinse and the placebo oral rinse arms, a sample size of 40 subjects per treatment group with probabilities of 80.5%, 87.1%, 91.6%, and 96.4% of observing an AE having underlying incidence probabilities of 4%, 5%, 6%, and 8%, respectively. The no-rinse control arm was considered to be of lesser importance and, thus, to conserve the overall sample size, was assigned a sample size of 20 subjects, which had probabilities of 55.8%, 64.2%, 71.0%, and 81.1% of observing an AE having underlying incidence probabilities of 4%, 5%, 6%, and 8%, respectively. Thus, the above sample sizes of 40, 40, and 20 subjects were expected to provide adequate power (> 80%) to detect AEs that occur at true underlying frequencies of 4% or greater in the test and placebo oral rinse study arms and adequate power (> 80%) to detect AEs that occur at true underlying frequencies of 8% or greater in the no-rinse control arm. To account for a 20% maximum attrition rate, total of 125 subjects were planned to be enrolled (50:50:25). Secondary efficacy analyses were considered as trend analysis with 70% power for detecting true differences between BLXA_4_ and placebo and 53% between no-rinse. The secondary endpoint analyses aimed to obtain estimates of the size and variance of possible treatment effects that can then be used to plan further clinical trials.

#### Statistical Analyses

Summary statistics of the secondary endpoints and their change from baseline, including n, means, minimums, maximums, standard deviations, and standard errors were summarized by study arm and study visit: MGI and BOP at Day 14 and Day 28. Exploratory analyses tested for treatment arm differences in the change from baseline of the exploratory efficacy outcomes: PD and CAL at study Day 28. Since the trend analysis was secondary, two different methods of analysis were used. Secondary endpoints were first analyzed using mixed models with changes from baseline as the dependent variable, subject as a random effect, a covariate adjustment for baseline, and fixed effects for visit, study arm, and a study arm-by-visit interaction. Then, as a *post-hoc* test, logistic regression analysis of generalized estimating equations (GEE) for ordinal measures were performed by using the log odds ratio to model the association between treatment arm pairs. Further, analysis of variance (ANOVA) for repeated measured used as *post-hoc* to test the site-specific changes in BOP over time between groups. Exploratory endpoints, PD and CAL, were analyzed using analysis of covariance (ANCOVA) with the PD or CAL change from baseline as the dependent variable, a covariate adjustment for baseline, and fixed effects study arm. The ANCOVA analyses assumed the outcomes were approximately normal in distribution with homogeneity of variance. Another exploratory endpoint, change in serum levels of lipid mediators were analyzed using principal component analysis (PCA) carried out using SIMCA software, version 13.0.3 (Umetrics, Umea, Sweden) (37). Changes in lipid mediator levels at Day 28 were also compared with baseline levels using one-way ANOVA followed by Tukey *post-hoc* test for multiple comparisons (IBM SPSS software version 19).

## Results

### Study Participants

**Figure 1:**
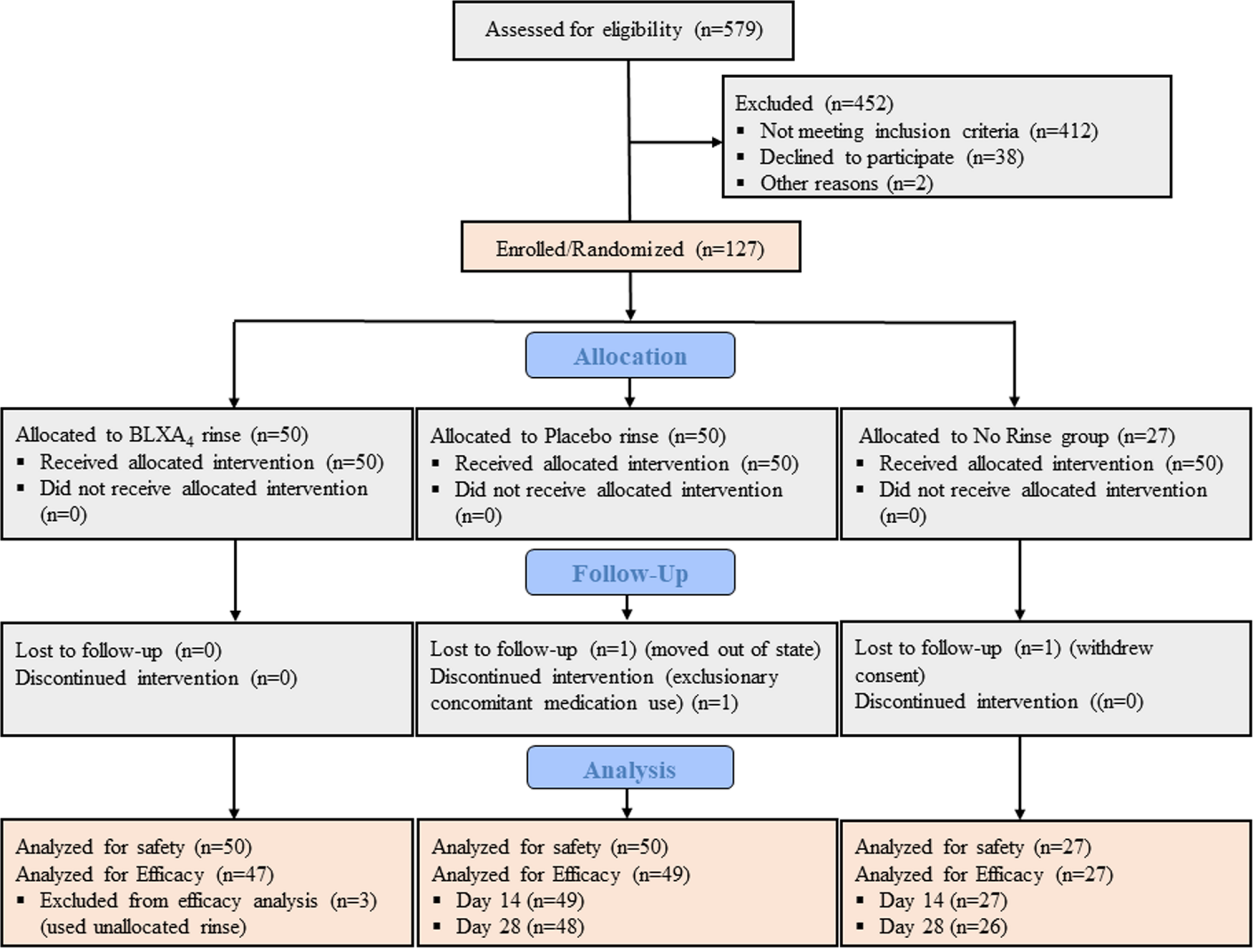
CONSORT diagram. CONSORT subject flow diagram shows the number of subjects screened, enrolled/randomized, and included in the primary safety and secondary efficacy analyses. Out of 579 patients screened, 127 individuals were found eligible, agreed to participate, and enrolled in the study. Fifty participants were randomized to BLXA_4_ rinse, 50 to placebo rinse and 27 participants to no rinse control group. The primary endpoint safety analysis included all subjects treated with at least one dose of BLXA4 or placebo or those who completed at least one follow up visit after baseline. The secondary endpoint efficacy analysis included those subjects who completed at least one follow up visit (starting at Day 14) for efficacy measures (PD, CAL, BOP, PI). Two subjects dropped out before the necessary Day 14 visit completion, thus replaced per protocol. In BLXA_4_ group, 3 subjects used unallocated rinse up to 3, 4 and 7 days respectively, thus excluded from efficacy analysis. 1 subject in placebo and 1 subject in no-rinse groups did not complete Day 28 visit (lost-to-follow up and withdrew consent, respectively) and excluded from efficacy analysis for that time point.

Between June 2015 and October 2017, total of 579 individuals were assessed for study eligibility, of those 127 were found eligible and randomized in one of the study groups constituting the safety population (**Figure 1**). Of these subjects, 50 were randomized to the BLXA_4_ rinse group, 50 to the placebo rinse group and 27 to the no-rinse control group. Participants (N=127) who received at least one dose of the study rinse or completed one post-baseline study visit were included in the primary endpoint analysis (safety). The efficacy population was composed of 123 participants who completed at least one post-baseline visit (starting at Day 14) for the assessment of efficacy outcome measures; participants who dropped out or withdrew prior to Day 14 were replaced in the efficacy population. In safety and efficacy populations, 122 (96.1%) and 121 (98.4%) study participants completed the study, respectively.

Baseline demographics are shown in **Table 2**. Most subjects in the safety population were male (62.2%), white (63.0%), and not Hispanic or Latino (78.0%). The median age was 45.0 years (range 18 to 65 years). Demographic characteristics were similar across the BLXA_4_, placebo, and no-rinse control groups with the exception of a greater proportion of men in the placebo group than in the BLXA_4_ group or no-rinse control group (BLXA_4_ 56.0%; placebo 70.0%; no-rinse control 59.3%), a greater proportion of black/African American subjects in the BLXA_4_ and no-rinse control groups than in the placebo group (BLXA_4_ 26.0%; placebo 16.0%; no-rinse control 25.9%), and a greater proportion of Hispanic or Latino subjects in both the BLXA_4_ or placebo groups than in the no-rinse control group (BLXA_4_ 24.0%; placebo 24.0%; no-rinse control 0%).

**Table 2:** Baseline clinical parameters (with 95% CI) and participant demographics by study group (safety and efficacy populations).

Demographics Characteristics	BLXA_4_	Placebo	Control (No rinse)	Overall
(Safety Population)	N=50	N=50	N=27	N=127
Sex - n (%)				
Male	28 (56.0)	35 (70.0)	16 (59.3)	79 (62.2)
Female	22 (44.0)	17 (30.0)	11 (40.7)	48 (37.8)
Age (Years)				
N	50	50	27	127
Mean	41.3	43.6	42.9	42.5
SD	14.35	12.88	12.16	13.27
Median	42	44	46	45
(Min, Max)	(19, 64)	(18, 65)	(19, 60)	(18, 65)
Race - n (%)				
American Indian/Alaska Native	1 (2.0)	0	0	1 (0.8)
Asian	4 (8.0)	2 (4.0)	3 (11.1)	9 (7.1)
Native Hawaiian or Other Pacific Islander	0	0	0	0
Black or African American	13 (26.0)	8 (16.0)	7 (25.9)	13 (22.0)
White	26 (52.0)	37 (74.0)	17 (63.0)	17 (63.0)
More Than One Race	3 (6.0)	1 (16.7)	0	4 (3.1)
Unknown or Not Reported	3 (6.0)	2 (4.0)	0	5 (3.9)
Ethnicity - n (%)				
Hispanic or Latino	12 (24.0)	12 (24.0)	0	24 (18.9)
Not Hispanic or Latino	36 (72.0)	36 (72.0)	27 (100)	99 (78.0)
Unknown of Not Reported	2 (4.0)	2 (4.0)	0	4 (3.1)
**Periodontal Parameters (Mean ± SD) (Efficacy Population)**	**N=47**	**N=49**	**N=27**	**N=123**
Modified Gingival Index (score of 0-4)	2.25 ± 0.1	2.30 ± 0.2	2.22 ± 0.2	2.26 ± 0.2
Bleeding on Probing (%)	43 ± 18	48 ± 19	39 ± 19	44 ± 19
Plaque Index (score of 0-3)	0.93 ± 0.5	0.94 ± 0.5	0.76 ± 0.5	0.90 ± 0.5
Pocket Depth (mm)	2.33 ± 0.3	2.41 ± 0.4	2.29 ± 0.3	2.36 ± 0.3
Clinical Attachment Level (mm)	1.50 ± 0.4	1.58 ± 0.4	1.40 ± 0.4	1.51 ± 0.4

Min, minimum; Max, maximum.

### Daily Rinsing With Mouthwash Containing 1.0µM BLXA_4_ Was Safe and Tolerated Well

All subjects tolerated the study treatment and study procedures well with only mild and temporary AEs possibly related to study. Thirty-two subjects reported a total of 56 treatment emergent adverse events (TEAEs), with 38% of subjects reporting at least 1 TEAE in the BLXA_4_ group, 20% of subjects in the placebo group (20.0%) and 11.1% subjects in the no-rinse control group with no statistically significant differences between groups. No TEAEs led to study drug discontinuation (**Table 3**). One SAE reported during the study by a participant in BLXA_4_ group was judged to be not study related.

**Table 3:** Primary endpoint, safety: summary of treatment emergent adverse event and event characteristics by study arm.

Treatment Emergent Adverse Event Characteristics	BLXA_4_ N=50		Placebo N=50		Control N=27	
	Number of Subjects n (%)	Number of Events n (%)	Number of Subjects n (%)	Number of Events n (%)	Number of Subjects n (%)	Number of Events n (%)
AEs	19	37	10	15	3	4
AEs by Severity						
Grade 1: Mild	18 (94.7)	35 (94.6)	10 (100)	15 (100)	3 (100)	4 (100)
Grade 2: Moderate	0	0	0	0	0	0
Grade 3: Severe or medically	1 (5.3)	1 (2.7)	0	0	0	0
significant but not immediately life- threatening						
Grade 4: Life-threatening consequences	1 (5.3)	1 (2.7)	0	0	0	0
Grade 5: Death related to AE	0	0	0	0	0	0
AEs by Relatedness to Study Drug						
Related^4^	6 (31.6)	8 (21.6)	4 (40.0)	5 (33.3)	0	0
Not Related^5^	15 (78.9)	29 (78.4)	6 (60.0)	10 (66.7)	3 (100)	4 (100)
AEs by Relatedness to Study Procedures						
Related^4^	4 (21.1)	5 (13.5)	1 (10.0)	1 (6.7)	0	0
Not Related^5^	17(89.5)	32 (86.5)	9 (90.0)	14 (93.3)	3 (100)	4 (100)
AEs that Led to Study Drug	0	0	0	0	0	0
Discontinuation						
AEs with an Outcome of Death	0	0	0	0	0	0

There were reports of transient oral TEAEs that were considered possibly related to study drug, including dry mouth reported once by 3 participants and an aphthous lesion reported by a single participant on a single occasion in the BLXA_4_ group. All oral TEAEs were rated as mild in severity and resolved without action. No clinically meaningful shifts in routine blood chemistry, hematology, urinalysis, oral ulceration or erythema of local tissues (Oral Mucositis Assessment Scale [OMAS]) were observed over time or among groups (**Supplementary Material**).

### Rinsing With a Mouthwash Containing BLXA_4_ Reduced Gingival Inflammation

**Figure 2:**
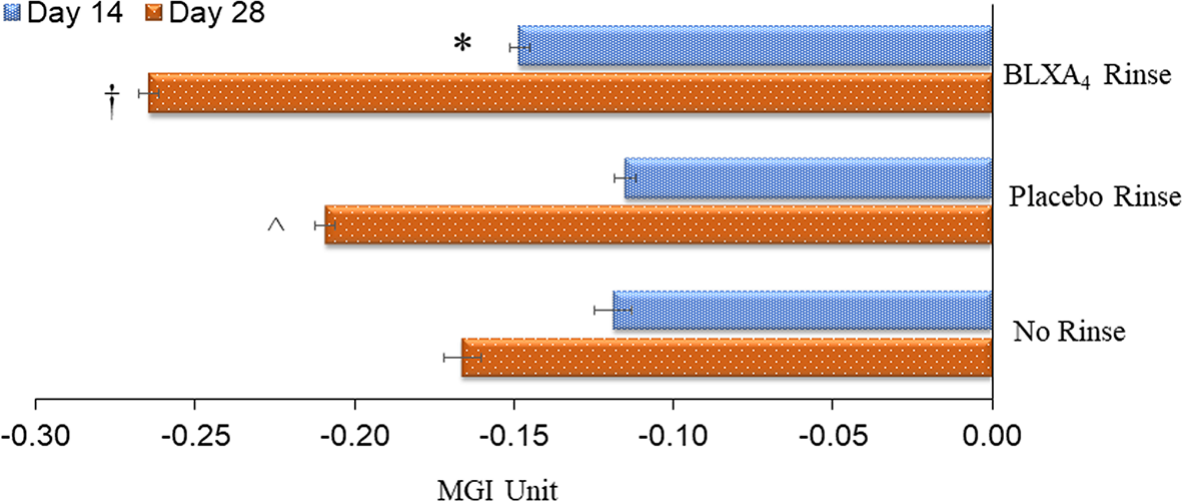
Change in gingival index: Primary efficacy endpoint. The key clinical endpoint associated with gingival inflammation was measured using the Modified Gingival Index (MGI) scored 0-4 (healthy-localized mild, generalized mild, moderate and severe). Change from baseline at Day 14 and Day 28 was compared between groups. The BLXA_4_ group demonstrated greater reduction in gingival inflammation compared to placebo and no rinse groups. *Significant difference compared to placebo rinse (p<0.05); †Significant difference compared to placebo rinse and no-rinse (p<0.05); ^Significant difference compared to no-rinse (p<0.05) by repeated measures mixed models analyses. N=123 (BLXA4: 47, Placebo: 49, No rinse: 27 subjects).

Once daily rinsing with the investigational mouthwash containing BLXA_4_ (1.0µM) resulted in a greater decrease in gingival inflammation measured by the modified gingival index (MGI) at Day 28 compared to placebo and no rinse control groups. In this analysis, two statistical methods were employed to explore trends. Regression analysis by least squares (LS) demonstrated a significant reduction in the mean MGI at Day 28 from baseline compared to the no-rinse control group (*P*=0.041) and a trend toward a difference compared to the placebo group (*P*=0.076) (**Figure 2**). Using generalized estimating equation (GEE) modelling for probability of having higher gingival index, we found that, compared to placebo rinse, BLXA_4_ rinse was significantly protective against increasing gingival inflammation over time (OR=0.75; 95% CI: 0.58 – 0.98; p=0.04).

**Figure 3:**
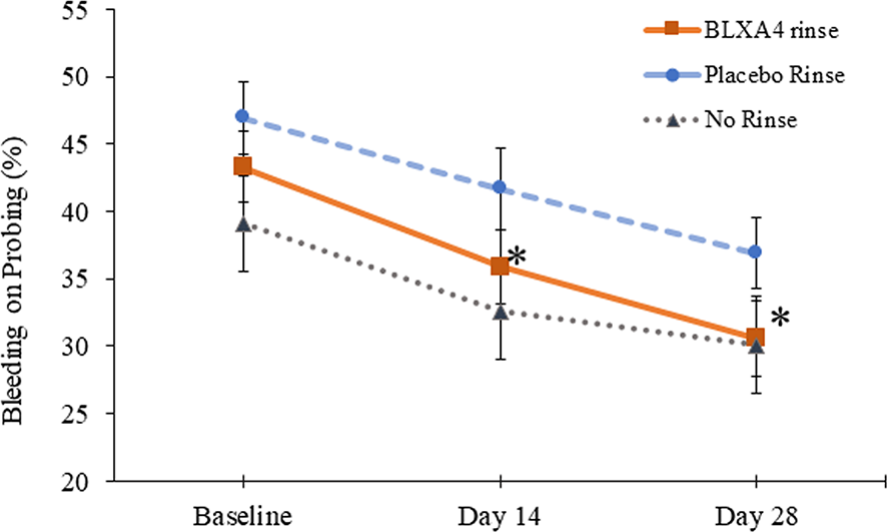
Change in bleeding on probing: Secondary efficacy endpoint. Another key clinical endpoint associated with gingival inflammation, bleeding on probing, was measured using a dichotomous measure, as 1=bleeding 0=no bleeding within 15 minutes following probing the site. Change from baseline at Day 14 and Day 28 was compared between groups. The BLXA_4_ group demonstrated greater reduction in gingival inflammation compared to placebo group at both Day 14 and Day 28 (*p<0.0001; *p=0.0053, respectively); ANOVA for repeated measures followed by Tukey-Kramer test. N=123 (BLXA4: 47, Placebo: 49, No rinse: 27 subjects).

Another key sign of gingival inflammation, gingival bleeding measured by percent of sites with bleeding on probing (BOP), decreased from baseline at both Day 14 and Day 28 in all three groups. Although the differences were not statistically significant based on regression analysis by least squares, reduction in BOP was steady and more pronounced in patients treated with BLXA_4_ rinse compared to placebo and no-rinse groups. The data was also analyzed *post-hoc* using ANOVA for repeated measures followed by Tukey-Kramer to test compare the site-specific changes over time compared with baseline. Percent reduction in bleeding on probing was statistically significant with BLXA_4_ treatment compared to changes seen with placebo at both Day 14 and Day 28 (*P*<0.0001 and *P*=0.0053) (**Figure 3**). There was a clear trend in differences between BLXA_4_ and the no-rinse group, however, they were not statistically significant (*P*=0.299 and *P*=0.111).

**Figure 4:**
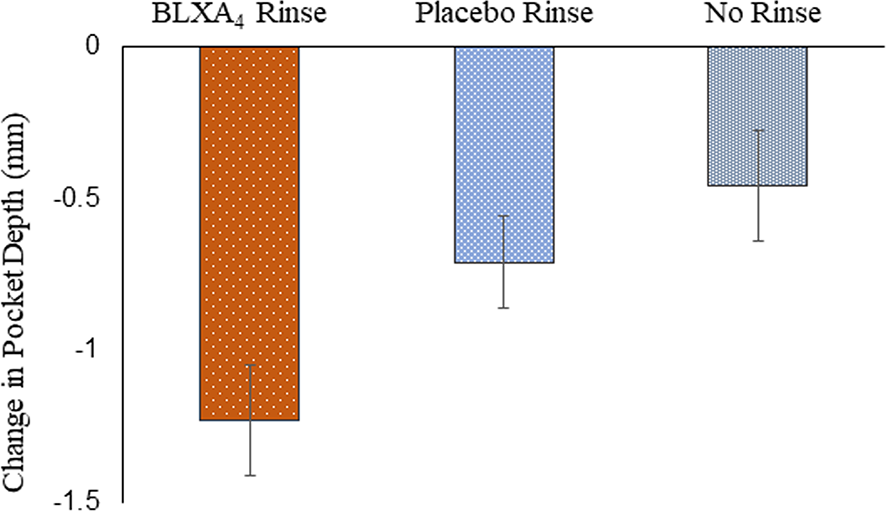
Change in probing depth: Secondary efficacy endpoint. A *post-hoc* analysis was conducted in a subgroup of subjects exhibiting periodontal disease with pocket depths ≥ 6 mm at baseline. There were 5 subjects in the BLXA4 group, 7 subjects in the placebo group and 5 subjects in the no rinse group. (BLXA4 *vs*. placebo, p=0.346; BLXA4 *vs*. no rinse, p=0.199) and (Placebo *vs*. no rinse p=0.639) by ANCOVA analysis. Note that all subjects had gingivitis with only a limited number of subjects exhibiting periodontal disease.

Analysis of additional secondary outcomes showed a clinically meaningful trends in the reduction of pocket depth with time. A sub-analysis of sites with baseline periodontitis (pocket depth ≥6 mm) and change over time revealed that periodontal pocket depth reduction (-1.23 ± 0.4 mm) with a 1.22 ± 0.6 mm clinical attachment gain was most pronounced in the BLXA_4_ group compared to placebo group (-0.71 ± 0.3 mm in PD with a CAL gain of 0.72 ± 0.3 mm) (**Figure 4**).

### Topical Oral BLXA_4_ Treatment of Gingival Inflammation Shifts the LM-SPM Profile From Proinflammatory to Pro-Resolution

**Figure 5:**
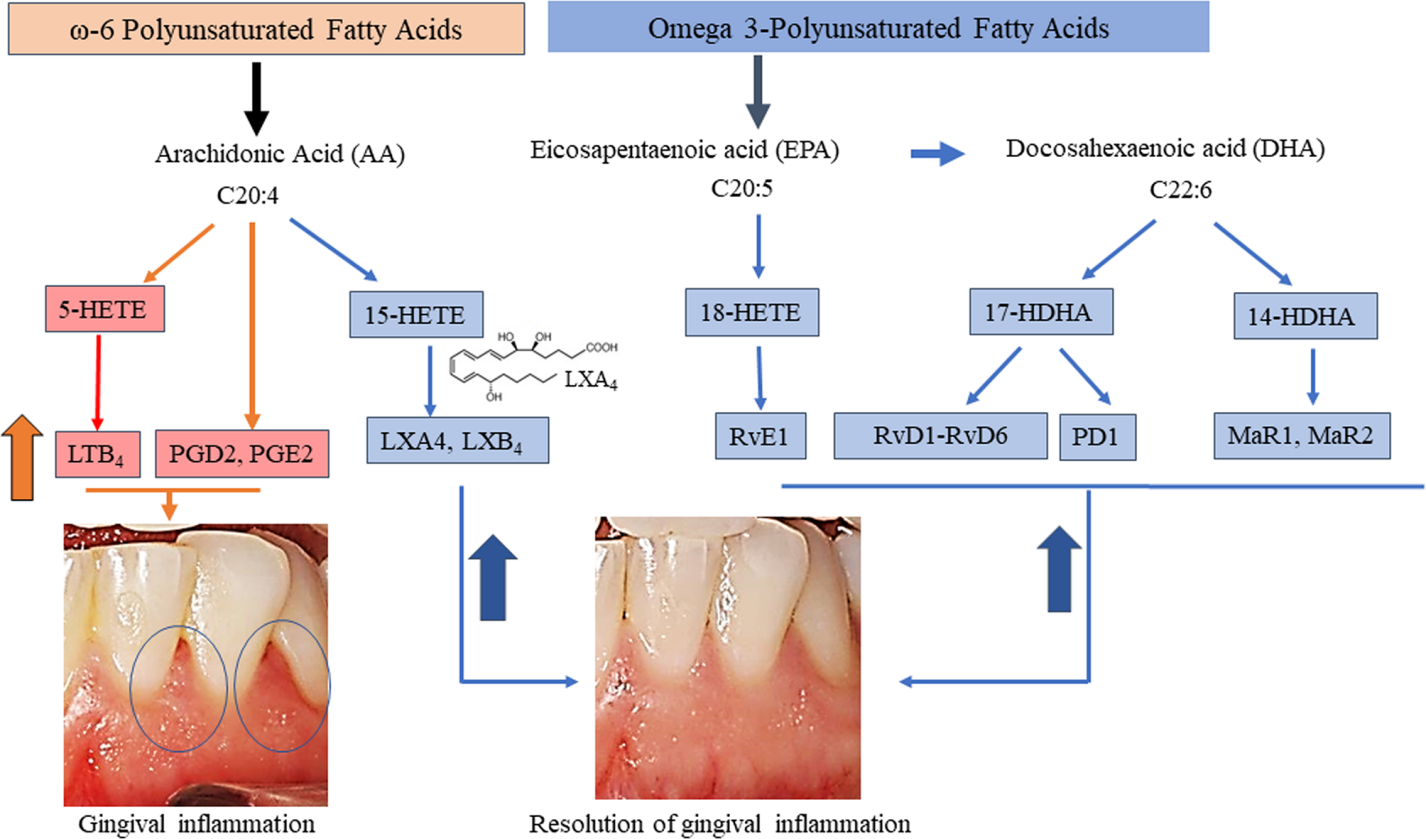
Overview of pathways of targeted eicosanoids and specialized pro-resolving mediators and resolution of gingival inflammation. Each are derived from three PUFA namely AA, EPA, and DHA with known biological functions of the pro-inflammatory mediators involved in the initiation of inflammation and of those involved in the resolution of inflammation are included. Mediators whose serum levels increased during inflammation (orange arrow and pink boxes) and during resolution of inflammation (blue arrow) following BLXA4 treatment on Day 28 are shown in blue shaded boxes, respectively.

**Figure 6:**
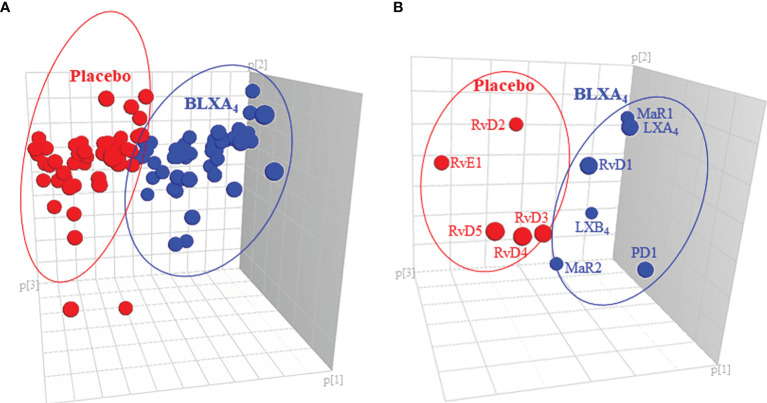
Impact of local BLXA_4_ treatment on the systemic inflammatory response. Quantitation and recovery of LMs in serum at baseline and at the end of the treatment on Day 28 were determined using the deuterium labeled internal standards (d8-5-HETE, d5-RvD2, d5-LXA4, d4-LTB4, d4-PGE2; d5-MaR2; 500 pg/μL), and a LM-SPM profile was constructed for each sample. Principal component analysis (PCA) was carried out to classify the observations (change in the BLXA_4_ and placebo groups) on the basis of lipid mediator levels. **(A)** 3D score plot of SPMs in serum at Day 28. Lipidomics profiles clearly separated the subjects treated with BLXA_4_ from placebo. **(B)** 3D loading plot of SPMs in serum at Day 28. Local treatment with BLXA_4_ significantly increased the abundance of SPMs in serum and distinctly separated the BLXA4-treated group from placebo group in the composition of lipid mediators indicating a shift in systemic inflammatory response toward resolution correlating with clinical findings.

For exploratory secondary biomarker endpoints, we analyzed the serum concentration of lipid mediator levels to determine the potential impact of local BLXA_4_ administration on overall systemic health. Serum levels of specialized pro-resolution lipid mediators (SPMs) were measured using targeted lipid mediator lipidomics monitoring 18 eicosanoids, SPMs and related pathway markers, including leukotriene B_4_ (LTB_4_), prostaglandins (PGD2, PGE2), lipoxins (9, 12-Benzo LXA_4_, LXA_4_, LXB_4_, LX pathway markers), E-series resolvins (RvE1), D-series resolvins (RvD1-RvD5), protectins (PD1) and maresins (MaR1, MaR2) (**Table 3** and **Figure 5**). All targets except BLXA_4_ were detected in all serum samples with varying levels. Principal component analysis (PCA) analysis was performed to classify the observations (change in the BLXA4 and placebo groups) on the basis of lipid mediator levels. Local treatment with topical BLXA_4_-ME markedly upregulated systemic production of several of the pro-resolving mediators known to orchestrate resolution of inflammation in animal models (**Figure 6A**). Notably, both arachidonic acid (ω-6) and ω-3 polyunsaturated fatty acid-derived mediators were increased, these included endogenous lipoxins (LXA_4_, LXB_4_) and resolvins (RvD1), protectins (PD1) and maresins (MaR1, MaR2), respectively, as shown in **Figure 6B** and **Table 4**. Local treatment with BLXA4 significantly increased the abundance of SPMs in serum and distinctly separated the BLXA4-treated group from placebo group in the composition of lipid mediators indicating a shift in systemic inflammatory response toward resolution correlating with clinical findings.

**Table 4:** Changes (Mean ± SE with 95% confidence intervals) at Day 28 from baseline (within group and between group comparisons for BLXA_4_ and placebo rinse groups).

Dependent Variable	(I) Group	(J) Group	Mean Difference (I-J)	Std. Error	Adjusted *p* value	95% Confidence Interval	
						Lower Bound	Upper Bound
PGD_2_	BLXA_4_-D28	BLXA_4_-BL	-32.78	8.16	**0.000**	-53.92	-11.64
		Placebo-D28	-25.14	8.16	**0.013**	-46.28	-4.00
	Placebo-D28	Placebo-BL	21.86	8.16	**0.040**	0.72	43.00
PGE_2_	BLXA_4_-D28	BLXA_4_-BL	-6.02	9.85	0.928	-31.55	19.51
		Placebo-D28	-4.79	9.85	0.962	-30.32	20.74
	Placebo-D28	Placebo-BL	3.52	9.85	0.984	-22.01	29.06
LTB_4_	BLXA_4_-D28	BLXA_4_-BL	-15.47	19.56	0.858	-66.16	35.22
		Placebo-D28	-22.23	19.56	0.667	-72.92	28.46
	Placebo-D28	Placebo-BL	4.94	19.56	0.994	-45.75	55.63
LXA_4_	BLXA_4_-D28	BLXA_4_-BL	0.97	0.27	**0.003**	0.27	1.67
		Placebo-D28	0.00	0.27	1.000	-0.70	0.71
	Placebo-D28	Placebo-BL	-0.82	0.27	**0.015**	-1.53	-0.12
LXB_4_	BLXA_4_-D28	BLXA_4_-BL	16.29	6.68	0.073	-1.02	33.60
		Placebo-D28	1.81	6.68	0.993	-15.50	19.12
	Placebo-D28	Placebo-BL	-4.02	6.68	0.931	-21.33	13.29
PD1	BLXA_4_-D28	BLXA_4_-BL	1024.69	310.33	**0.006**	220.26	1829.12
		Placebo-D28	177.73	310.33	0.940	-626.70	982.15
	Placebo-D28	Placebo-BL	863.50	310.33	0.116	59.07	1667.93
MaR1	BLXA4-D28	BLXA_4_-BL	68.60	33.57	0.176	-18.42	155.62
		Placebo-D28	63.75	33.57	0.232	-23.27	150.77
	Placebo-D28	Placebo-BL	6.58	33.57	0.997	-80.44	93.60
MaR2	BLXA_4_-D28	BLXA_4_-BL	1.91	2.40	0.858	-4.33	8.14
		Placebo-D28	4.31	2.40	0.279	-1.92	10.55
	Placebo-D28	Placebo-BL	-3.54	2.40	0.457	-9.77	2.69
	BLXA_4_-D28	BLXA_4_-BL	8.96	4.51	0.196	-2.72	20.65
RvD1		Placebo-D28	3.98	4.51	0.814	-7.71	15.66
	Placebo-D28	Placebo-BL	1.71	4.51	0.981	-9.97	13.39
RvD2	BLXA_4_-D28	BLXA_4_-BL	2.29	1.61	0.487	-1.88	6.47
		Placebo-D28	3.23	1.61	0.191	-0.95	7.40
	Placebo-D28	Placebo-BL	-3.42	1.61	0.150	-7.60	0.76
RvD3	BLXA_4_-D28	BLXA_4_-BL	7.25	3.87	0.243	-2.78	17.28
		Placebo-D28	5.71	3.87	0.455	-4.32	15.74
	Placebo-D28	Placebo-BL	-3.55	3.87	0.795	-13.58	6.48
RvD4	BLXA_4_-D28	BLXA_4_-BL	2712.73	851.93	**0.009**	504.40	4921.07
		Placebo-D28	612.78	851.93	0.102	-1595.56	2821.11
	Placebo-D28	Placebo-BL	-2570.95	851.93	**0.015**	-4779.29	-362.62
RvD5	BLXA_4_-D28	BLXA_4_-BL	9.72	2.54	**0.001**	3.14	16.31
		Placebo-D28	0.76	2.54	0.991	-5.82	7.34
	Placebo-D28	Placebo-BL	7.58	2.54	0.117	1.00	14.16
RvE1	BLXA_4_-D28	BLXA_4_-BL	3.41	32.19	0.983	-80.04	86.86
		Placebo-D28	55.44	32.19	0.315	-28.01	138.88
	Placebo-D28	Placebo-BL	-10.62	32.19	0.988	-94.07	72.82
HDHA14	BLXA_4_-D28	BLXA_4_-BL	107.55	99.86	0.704	-151.29	366.40
		Placebo-D28	55.17	99.86	0.946	-203.68	314.01
	Placebo-D28	Placebo-BL	-48.34	99.86	0.963	-307.18	210.51
HDHA17	BLXA_4_-D28	BLXA_4_-BL	20.90	99.11	0.997	-236.00	277.79
		Placebo-D28	14.45	99.11	0.999	-242.44	271.35
	Placebo-D28	Placebo-BL	6.44	99.11	1.000	-250.46	263.34
HEPE18	BLXA_4_-D28	BLXA_4_-BL	34.23	15.23	0.114	-5.25	73.70
		Placebo-D28	18.86	15.23	0.603	-20.62	58.33
	Placebo-D28	Placebo-BL	-22.21	15.23	0.465	-61.68	17.27
HETE 15	BLXA_4_-D28	BLXA_4_-BL	12.58	38.60	0.988	-87.46	112.62
		Placebo-D28	24.32	39.00	0.924	-76.75	125.40
	Placebo-D28	Placebo-BL	-45.83	39.40	0.651	-147.93	56.28

Bold **p values** denote significance at the 0.05 level.

A negative mean value indicates an increase in the level of lipid mediator and a positive value denotes a decrease in the level of lipid mediator at Day 28 compared to baseline. ANOVA followed by a post hoc test (Tukey’s test) for multiple comparisons.

Red color indicates a noteworthy decrease from baseline, where indicated, while blue denotes a noteworthy increase from baseline at p<0.05.

Orange shaded box lists pro-inflammatory lipid mediators, PGD_2_, PGE_2_ and LTB_4_.

Gray shaded box lists omega 6 lipoxygenase pathway pro-resolution agonist, Lipoxin A_4_ and LXB_4_.

Yellow shaded box lists DHA-derived specialized pro-resolving mediators (D-series resolvins, protectins, maresins).

Blue shaded box lists EPA-derived specialized pro-resolving mediator, resolvin E1.

Unshaded box lists precursors of lipoxins, D series and E series resolvins (Rv), protectins (PD) and maresins (MaR).

**Figure 7:**
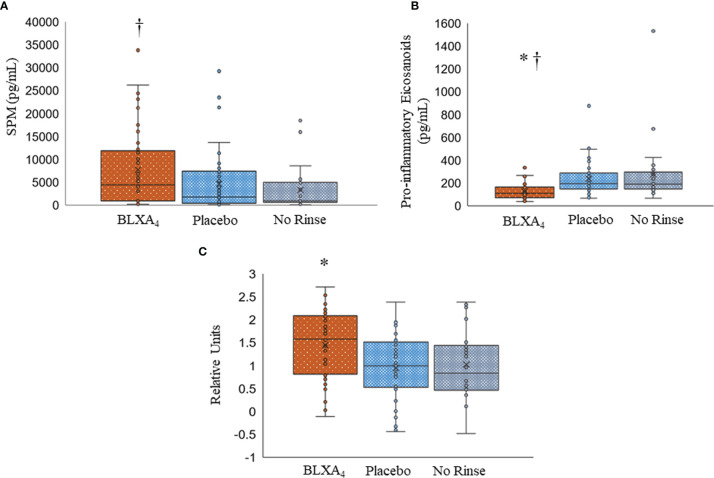
Changes in serum lipid mediator profiles by topical BLXA_4_-ME treatment. **(A)** Sum of pro-resolving mediators (n-3 DHA-derived RvD1-6, PD1, MaR1, MaR2, n-3 EPA-derived RvE1, and AA-derived LXA_4_, LXB_4_). **(B)** Sum of proinflammatory eicosanoids (AA-derived LTB_4_, PGE_2_ and PGD_2_). Results are means ± 95% confidence interval for each subject. **(C)** Log_10_ ratio of pro-resolving mediators (SPMs) to proinflammatory eicosanoids, PGD_2_, PGE_2_ and LTB_4_ (Resolution Index**)**. The ratio of pro-resolving lipid mediators to proinflammatory eicosanoids at Day 28 was calculated to determine the resolution index after treatment with BLXA_4_. Results are shown as individual results for each patient, horizontal bars depicting median values. Statistical analysis was conducted using one-way ANOVA followed by Tukey *post-hoc* test. † p<0.05 compared to no rinse; * p<0.05 compared to placebo; N=47 BLXA_4_-treated subjects, N=48 placebo and N=26 no rinse.

We next assessed the LM profiles of serum from BLXA-treated study group and serum from placebo group by grouping the mediators based on their biologic functions, comparing the differences between pro-resolving mediators (LXs, resolvins, protectins, and maresins) and proinflammatory mediators LTB4, PGE2, and PGD2 (**Figure 7A** and **Table 4**). Results of this analysis demonstrated an increase in overall pro-resolving lipid mediator concentrations in the serum of BLXA4-treated individuals, while there was a decrease in pro-inflammatory mediator concentrations compared to placebo group (*P*=0.08 and *P*=0.004, respectively) and no rinse group (p=0.027 and p=0.001), respectively) (**Figure 7B** and **Table 4**). The changes in SPM and pro-inflammatory mediator profiles are also supported by an overall pro-resolution lipid mediator profile measured by an increase in the resolution index (*P*=0.003 compared to placebo and *P*=0.056 compared to no-rinse). Resolution index was calculated by dividing the sum of the resolution mediators by the sum of proinflammatory eicosanoids for each subject (**Figure 7C**) (37).

No significant differences were observed in post-treatment supragingival plaque levels or IL-1β levels in the gingival crevicular fluid between BLXA_4_-treated, placebo-treated or no-rinse control groups (data not shown).

## Discussion

This is the first human oral treatment study to examine BLXA_4_ oral rinse as a potential new therapy in patients with periodontal inflammation. We show that topical administration of a pro-resolving lipid mediator administered through a daily mouthwash is feasible, well-tolerated and effective to reduce oral inflammation. In addition, we document the first eicosanoid-SPM signatures in peripheral blood serum markedly upregulated as a result of a 28-day oral topical application of BLXA_4_. Safety was assessed using transient emerging adverse events over the course of the trial as the primary outcome. There were no adverse safety signals noted throughout the trial. The few adverse events reported were not severe and the only potentially related event to the mouth rinse was mild oral irritation, which was distributed evenly between groups. Oral irritation was likely due to sodium lauryl sulfate in the mouth rinse preparation.

Lipoxins are a class of endogenously biosynthesized mediators that promote the resolution of inflammation (38). Biological actions of lipoxins include limiting neutrophil infiltration, promotion of macrophage polarization, increase of macrophage efferocytosis, and restoration of tissue homeostasis (39). The mechanism includes down-regulation of pro-inflammatory cytokines and chemokines, inhibition of the activation of the master pro-inflammatory pathways and increased release of pro-resolving cytokines (e.g., interleukin-10) (39). Accumulating evidence from our laboratory (29, 40–42) and many others (24, 43–48) has demonstrated that lipoxins and synthetic analogues protect tissues from damage from excess inflammation and reverse chronic inflammation promoting return to tissue homeostasis. We demonstrated earlier in animal models of periodontitis that oral topical application of lipoxin A_4_ (29) and resolvin E1 (26–28, 49) significantly protect against soft tissue and alveolar bone destruction caused by local periodontal inflammation, and induce regeneration of destroyed periodontal tissues, including gingiva, periodontal ligament and alveolar bone. In addition to local inflammation, topical application of LXA_4_ and RvE1 significantly dampened the systemic inflammatory burden as demonstrated by reduced levels of serum C-reactive protein and interleukin 1β (26, 29, 49).

Periodontal disease is a bacterial biofilm-induced chronic inflammatory disease resulting in loss of connective tissue attachment to the teeth and osteoclast-mediated alveolar bone resorption (50). Leukocytes play multiple roles in the progression of periodontal disease, including phagocytosis and killing of bacteria, secretion of inflammatory cytokines, mounting of specific immune response and activation of osteoclasts (51). Leukocytes are essential in the host defense against oral pathogens; however, in susceptible individuals unable to resolve the inflammatory lesion, chronic inflammation in the periodontium causes periodontal bone loss (52)

This phase 1 clinical trial is the first human study to evaluate the therapeutic opportunity provided by lipoxin A_4_ as a novel approach to treat chronic oral inflammatory disorders, focusing on periodontal disease. Periodontitis is an example of a host leukocyte-mediated disease. Current periodontal therapy focuses on control of the bacterial load, but these approaches have limited success and disease recurrence is a frequent problem. Moreover, periodontal tissue regeneration is not possible with antimicrobial approaches alone. The promise shown for host modulating approaches that regulate inflammatory immune responses without pharmacologic inhibition of pro-inflammatory pathways that are associated with significant side-effects prompted drug development in this area.

The phase1 clinical trial was powered for safety, and efficacy variables were evaluated secondarily. While underpowered, examination of changes in gingival inflammation revealed that BLXA_4_ topically applied in a mouthwash to inflamed oral/periodontal tissues demonstrated significant clinical benefits. Gingival inflammation measured with two separate methods (modified gingival index and bleeding on probing) was markedly reduced as was pocket depth. To gain insight into the impact of treatment on periodontitis, a sub-analysis of a small set of subjects who exhibited clinical periodontitis was performed. Subjects with pocket depth ≥6 mm showed a greater reduction (-1.23 ± 0.4 mm) with a 1.22 ± 0.6 mm clinical attachment gain in the BLXA_4_ group compared to placebo group (-0.71 ± 0.3 mm in PD with a CAL gain of 0.72 ± 0.3 mm). This is a particularly interesting clinical finding as with conventional and robust therapeutic approaches comprising standard of care (scaling and root planning in conjunction with systemic antibiotics), the clinically significant average pocket depth reduction is -0.86 mm with a clinical attachment gain of 0.75 mm (53). Reduction in gingival index, bleeding on probing and periodontal pocket depth suggest that BLXA_4_ is a promising non-invasive therapeutic approach for the treatment of inflammatory periodontal diseases.

Based on these earlier pre-clinical animal results, we establish here, for the first time in this phase 1 clinical trial that BLXA_4_ at a 1.0µM (284 µg/ml) dose (the minimal effective dose previously shown to have an impact in a rabbit model of periodontitis) demonstrates significant clinical potential in increasing the endogenous levels of SPMs critical for a strong protective and pro-resolving innate immune response. In particular, specific SPMs including lipoxins (LXA_4_, LXB_4_), resolvins (RvE1, RvD1-RvD6), protectins (PD1) and maresins (MaR1, MaR2) responsible for a range of cellular and biochemical events in resolution of inflammation, and their precursors (15-HETE, 18-HEPE, 17-HDHA), were differentially expressed in the serum of BLXA_4_-treated participants. These findings are consistent with earlier results from animal models suggesting the potential of pro-resolving agonists for treating oral/periodontal diseases associated with infection, inflammation, and altered phagocyte functions (3, 26–28, 54). More importantly, the robust actions of topically applied BLXA_4_ on systemic levels of SPMs suggest that oral tissues can serve as an easy-to-access application route for potential treatment of a wide range of inflammatory diseases (55–58). BLXA_4_ was not detected, likely due to its clearance to below detectable levels after administration. Pharmacokinetic studies planned for in the Phase 2 trial are necessary to determine the kinetics of BLXA_4_ in circulation.

As a Phase 1 trial, this study has a few shortcomings. First, the study was primarily conducted for testing the safety of BLXA_4_ in humans; thus, the study was underpowered for detecting the differences in clinical efficacy endpoints. Second, the study included a group of subjects (no-rinse group) that did not use any rinse aiming to detect the confounding impact of the mechanical actions of rinsing. This no rinse group could, therefore, not be double blinded. In addition, the study assumed the effective dose of the BLXA_4_ based only on studies in an animal model of periodontal inflammation, thus the effect size of the BLXA_4_ compared to placebo could be underestimated. Lastly, the study was not precisely powered to rule out the potential Hawthorne effect (59, 60) (performing better once in a clinical trial regardless of the assigned study group) in clinical trials. It is, however, worth mentioning that despite a small sample size and limited power, the study was large enough to detect an impact on the key inflammatory parameters, GI and BOP, and systemic levels of pro-resolution lipid mediators at the minimal effective dose of BLXA_4_ determined in animal trials. Further, finding a trend in a greater pocket depth reduction in a subgroup of subjects having periodontitis with deeper pockets in such a short time frame (28 days) highlights the potential role of BLXA_4_ in the treatment of periodontal diseases. In addition, the duration of 28 days could be a limiting factor to determine long-term effects, primarily the impact on compliance. Nevertheless, further dose-response studies powered for testing clinically meaningful differences in clinical periodontal endpoints are needed.

Taken together, in this Phase 1/2 randomized controlled clinical trial, rinsing with a potent BLXA_4_ containing mouthwash was well tolerated and presented no safety concerns. No safety signals beyond mild oral irritation were identified. Side effects, measured as transient emerging adverse events, were rare, mild and not associated with the drug. Topical application of BLXA_4_ with a once-daily mouth rinse for 28 days effectively reduced gingival inflammation without additional mechanical treatment supporting the power of host-modulatory therapies. Remarkably, the study revealed, for the first time, that topical application of BLXA_4_, beyond its local impact, shifted lipid mediators in serum toward a pro-resolution profile with significant changes in SPM levels compared to placebo. This finding demonstrates the potency of BLXA_4_ at a topically applied micromolar dose that is effective in systemic regulation of resolution pathways as we found increases in specific serum SPMs in the individuals treated with BLXA_4_. BLXA_4_ applied to human local oral tissues in a randomized trial was capable of shifting the inflammatory balance to resolution in periodontal inflammation with potential applications for other inflammatory diseases. Further, the stability of BLXA_4_ in aqueous solutions (at least 3 months at room temperature) makes it suitable for further development as a therapeutic agent.

## Data Availability Statement

The data that support the findings of this study are available from the corresponding author upon reasonable request.

## Ethics Statement

The studies involving human participants were reviewed and approved by Forsyth Institute Institutional Review Board. The patients/participants provided their written informed consent to participate in this study. The animal study was reviewed and approved by Calvert Laboratories.

## Author Contributions

HH and TD designed and conducted the clinical trial as the principal investigator and sponsor for the FDA IND, respectively. HH and TD analyzed and interpreted the data, wrote, and critically revised the paper. HH, MM, HS, CF, and MC performed clinical trial. FS and MH conducted lipidomics analysis, C-JC performed *post-hoc* statistical analyses for clinical endpoints. All authors contributed to the article and approved the submitted version.

## Funding

This work was supported by the NIH/NIDCR grants PO1 DE13499, P50 DE016191, and U01 DE016191.

## Acknowledgments

We thank The Forsyth Institute Center for Clinical and Translational Research clinician and administrative team, Elida Salazar, Gay Torresyap, Marie Letteri, Molly Dunne, laboratory technicians Daniel Nguyen, Danielle Stephens and Michele Patel for technical support and collaborators at Brigham and Women’s Hospital, Dr. Charles N. Serhan for valuable contribution to BLXA_4_ design and manufacturing, preclinical studies and interpretation of lipidomics data and Charlotte Jouvene for help with PCA analyses. We also thank Dr. Katherine Lemon for help in the assessment of safety laboratory tests as well as Heather Jared and the clinical research team at Rho, Inc., for administrative, regulatory and analytical assistance. The expertise and efforts of Drs. Walt Shaw and Steve Burgess (Avanti Polar Lipids, Inc.) and their team for the synthesis and production of the GMP mouth rinse and placebo used in this study are gratefully acknowledged. We also thank the study participants for time and commitment during the study and Dr. R. Dwayne Lunsford and the team at NIDCR-NIH for help in each step needed prior to starting this human study. Supported by the National Institute of Dental and Craniofacial Research grants (Dr. Charles N. Serhan and TD, Grants P50 DE016191 and PO1 DE13499, and TD and HH, Grant U01 DE016191).

## Conflict of Interest

HH and TD are inventors on several granted and pending licensed and unlicensed patents awarded to the Forsyth Institute that are subject to consulting fees and royalty payments.

The remaining authors declare that the research was conducted in the absence of any commercial or financial relationships that could be construed as a potential conflict of interest.

## Publisher’s Note

All claims expressed in this article are solely those of the authors and do not necessarily represent those of their affiliated organizations, or those of the publisher, the editors and the reviewers. Any product that may be evaluated in this article, or claim that may be made by its manufacturer, is not guaranteed or endorsed by the publisher.
