# Supplementary material for: Safety and Preliminary Efficacy of a Novel Host-Modulatory Therapy for Reducing Gingival Inflammation
Source: Front Immunol. 2021 Sep 13;12:704163. doi: 10.3389/fimmu.2021.704163 (PMC8475270; doi:10.3389/fimmu.2021.704163)
Supplement: Supplementary file 1 [file DataSheet_1.docx]

Supplementary Material

1. **Supplementary Data**

**1.1. Materials and Methods**

***Clinical safety assessments***

A complete medical history was obtained at the Screening Visit. The medical history also included demographic information, alcohol and tobacco history, medications, dental procedures, and dental history information.

Vital signs, including oral temperature, pulse rate, respiratory rate, and blood pressure, were measured at all study visits beginning with the Screening Visit and through the Day 28 Visit. Weight was collected only at the Screening and Day 28 Visits.

An intraoral and extraoral examination was conducted at each visit from baseline through the Day 28 Visit. During the intraoral examination, the oral cavity was evaluated for ulceration and erythema using the Oral Mucositis Assessment Scale (OMAS) (1). The first 10 subjects were scored by 2 independent examiners at each study visit through Day 28 to ensure consistency across examiners. After the first 10 subjects, the OMAS was scored only once at each study visit. The OMAS was used to evaluate ulceration and erythema at the following sites: Upper and lower lips; hard and soft palate/fauces; right and left buccal mucosa; ventral and lateral surfaces on the tongue; floor of mouth.

Adverse events were recorded at each visit except the screening visit and baseline visit. The participant provided the completed diary, and was interviewed to collect information regarding solicited AEs.

Blood and urine samples were sent to a clinical laboratory (Quest Diagnostics Laboratories, Marlborough, MA) for the tests including complete blood count [hemoglobin, hematocrit, red blood cell count (RBC), white blood cell (WBC) count with differential and absolute counts by WBC type, platelet count, and RBC indices (mean corpuscular volume, mean corpuscular hemoglobin concentration, RBC distribution width)], erythrocyte sedimentation rate, serum chemistry tests including creatinine, blood urea nitrogen, alkaline phosphatase, AST, ALT, total bilirubin, and electrolytes (sodium, potassium, chloride, and bicarbonate) and complete urinalysis including color, appearance, specific gravity, pH, glucose, bilirubin, ketones, occult blood, protein, nitrite, leukocyte esterase, WBCs, RBCs, squamous and other epithelial cells, bacteria, casts, and crystals.

Subjects with serum creatinine values greater than 50% above their baseline level were retested, even if within normal range. Subjects with persistently abnormal values at the retest were referred to their primary care physician and were followed until the values returned to the normal range or were confirmed as stable with an adequate explanation. Additionally, the on-site medical monitor reviewed the laboratory results to assist the principal investigator with assessing and monitoring AEs.

Supragingival plaque samples were obtained at the baseline visit, Day 14, and Day 28 and analyzed for any increase in opportunistic or pathogenic bacteria. Supragingival plaque was obtained from 6 sites in the mouth (the mesiobuccal surfaces of the Ramfjord teeth). Samples were placed in transport media for DNA checkerboard probe analysis (DNA-DNA hybridization assay) and analyzed according to the method of Socransky (2, 3). Samples were analyzed for the presence of *Fusobacterium nucleatum* subspecies *vincentii,* *Campylobacter concisus, Campylobacter rectus, Tannerella forsythensis, Prevotella intermedia, Prevotella nigrescens, Porphyromonas gingivalis, Capnocytophaga sputigena, Streptococcus oralis, Actinomyces naeslundii, Actinomyces israelii, Eubacterium brachy, Eikenella corrodens,* and *Treponema denticola*.

***Physical and chemical properties, structural and molecular formulas and molecular weight of BLXA_4_***

The chemical name of the investigational drug substance (currently known as BLXA_4_-ME, previously known as 9,12-LXA_4_) is (5S, 6R, E)-methyl-5,6-dihydroxy-8-(2-((R,E)-3-hydroxyoct-1-enyl) phenyl) oct-7-enoate. BLXA_4_ features a substituted benzo-fused ring system at the o-[9, 12] position, replacing the tetraene unit of native lipoxin-A_4_. This structural feature imparts both chemical and metabolic stability to the molecule while retaining potent biological activity. The molecular formula for BLXA_4_ is C_23_H_34_O_5_ and its molecular weight is 390.51g/mole.

The oral rinse formulation containing the active compound and placebo rinse were prepared by Avanti Polar Lipids, Inc. Alabaster, Alabama, in a facility that adheres to Good Manufacturing Practice. The active drug substance, BLXA_4_, is a white to off-white powder. The formulated 1µM BLXA_4_ mouth rinse, namely ClinRinse-1, was a homogeneous suspension of BLXA_4_ in aqueous vehicle solution containing the inactive components listed in Table S1; it was a clear liquid with peppermint scent. The inactive components of the oral rinse comprised >99.9% of the drug product. To achieve a 1.0 μM solution of drug product, the manufacturer added 390.51μg of BLXA_4_ drug substance per liter of vehicle.

The formulated BLXA_4_ mouth rinse and placebo rinse were packaged in opaque high density polyethylene narrow mouth cylindrical bottles, containing 255 mL of rinse. The closure system was a threaded polypropylene lid with a groove facilitating the seal. Bottles were over-sealed with 30.5 mm x 25 mm opaque white cellulose tamper-evident bands.

The BLXA_4_ mouth rinse or placebo rinse was provided to study participants in the original bottles supplied by the manufacturer. A 30 mL polypropylene dosing cup with volume markings was provided with the rinse. Each bottle of study product contained a volume sufficient for 8 doses plus an excess volume to accommodate for an out-of-window study visit. The residual volume was measured to assess compliance with product use. Labels on the rinse bottles complied with the regulations for labeling of an investigational product (21 CFR Part 312.6), and the active and placebo rinses were identified by codes to maintain the blinding of subjects and investigators.

The investigational product was stored at 2 to 8°C at the clinical study site. Study participants were provided a bottle containing an 8-day supply of oral rinse; during the period of use (approximately 1 week), the rinse was stored in the original bottle at room temperature (range 15 to 30°C, 59 to 86°F), to facilitate compliance with the daily use protocol.

Testing of the drug product demonstrated that it was stable at 2 to 8°C for up to 12 months, and stable at 25°C/60% relative humidity for up to 4 weeks (Batch Production Records, Avanti Polar Lipids, Inc.).

***Pre-clinical studies******: safety pharmacology and toxicology***

BLXA_4_ was evaluated *in vitro* for its potential to block the inward-rectifying hERG potassium channel, using human embryonic kidney cells that express hERG. BLXA_4_ was also evaluated for potential adverse pharmacological effects in vivo. These studies are summarized in Table S2. All preclinical studies were conducted at a commercial laboratory complied with Good Laboratory Practices (Calvert Laboratories, Inc. Scott Township, PA).

The half-maximal inhibitory concentration (IC50) for the inhibitory effect of BLXA_4_ on hERG potassium current was estimated to be greater than 10 μM, a concentration that is at or above the plasma concentrations achieved in dogs administered an oral gavage dose of 2000 μg/kg. No adverse cardiovascular findings were observed in dogs following oral administration of BLXA_4_ at doses up to 2000 μg/kg, and the plasma concentration of BLXA_4_ achieved at this dose was approximately 150 to 225 times that anticipated to be achieved in humans if a research subject swallowed the 30 mL clinical rinse dose containing 11.7 μg BLXA_4_.

***Pharmacokinetics and drug metabolism in animals***

Under physiological conditions, BLXA_4_-ME is converted by nonspecific esterase activity to a free acid (FA) form designated as BLXA_4_-FA. Both the ME and FA forms of BLXA_4_ were pharmacologically active.

***Absorption***

To determine potential systemic exposure following unintended exposure by the oral route (swallowing of oral rinse), the pharmacokinetics of BLXA_4_-ME were evaluated following administration of a single oral dose in rats (15 males and 15 females) and beagle dogs (4 males and 4 females).

Following a 2000 μg/kg dose, levels of parent compound BLXA_4_-ME in rat plasma were low to nondetectable; however, the kinetics of the primary free acid metabolite, BLXA_4_-FA, were established in rat plasma, and are shown in Table S3. Similarly, the kinetics of BLXA_4_-ME were established in male and female beagle dogs following a single oral dose of 2000 μg/kg, as shown in Table S4. Parent compound BLXA_4_-ME persisted for somewhat longer in dogs than in rats, although overall levels of both ME and FA forms were generally lower in dogs compared with rats at the same dose level. Exposure to both BLXA_4_-ME and BLXA_4_-FA appeared to be slightly greater in female than in male dogs.

***Metabolism***

In vitro studies have established that rat microsomes rapidly catalyze nearly complete conversion of BLXA_4_-ME to BLXA_4_-FA, whereas both human and dog microsomes are much slower in converting the ME to the FA form. Similarly, rat plasma was much more effective than either human or dog plasma in metabolizing BLXA_4_-ME to BLXA_4_-FA. In addition, BLXA4-ME was notably protein-bound in human and dog plasma, and was entirely metabolized to the FA form in rat plasma.

At concentrations up to 15 μg/mL, BLXA_4_-ME did not appear to significantly inhibit human liver cytochrome P450 enzymes (CYP) in vitro. Of the CYP isoforms, only CYP2C9 and CYP2C19 showed inhibition (IC50 of 12 and 4 μg/mL, respectively).

***Toxicology***

Single dose studies: Preliminary studies in rodents were undertaken to establish the safety, tolerability, and exposure to BLXA_4_-ME following acute dose exposure via oral gavage. BLXA_4_-ME was well tolerated as an acute dose up to 1000 μg/kg in rats (30 males and 30 females) and systemic exposure was established at very early time points following dosing. These early rodent studies revealed that the BLXA_4_-ME (methyl ester) form of the product was rapidly converted in vivo in the rats to the BLXA_4_-FA (free acid) form of the molecule, which was still pharmacologically active.

BLXA_4_-ME at a dose of 2000 μg/kg was also well tolerated following acute dosing via oral gavage in 4 male and 4 female beagle dogs, and plasma levels of nearly 1 μg/mL were achieved within 30 minutes of dosing, suggesting rapid absorption from the gastrointestinal tract in dogs. No toxicity was observed.

*Repeat dose studies*: To assess whether BLXA_4_-ME would accumulate with repeated dosing, 7-day and 28-day oral exposure studies were conducted in both rats and beagle dogs, with doses up to 2000 μg/kg/day delivered via oral gavage.

For the 7-day repeat dose study, toxicity was assessed in groups of 5 male and 5 female rats per dose level and toxicokinetic assessments were performed in groups of 9 male and 9 female rats per dose level. For the 28-day repeat dose study, toxicity was assessed in groups of 10 male and 10 female rats for low and mid-dose levels (200 and 500 μg/kg/day) and in 15 male and 15 female rats for the high dose (2000 μg/kg/day); toxicokinetic assessments were performed in groups of 9 male and 9 female rats for each dose level.

The 7-day repeat dose toxicity study in beagle dogs included one male and one female per dose level. The 28-day repeat dose toxicity and toxicokinetic study included 3 males and 3 females in the low and mid-dose groups (200 and 500 μg/kg/day), and 5 males and 5 females in the high dose group (2000 μg/kg/day).

While significant systemic exposure was achieved in both rats and dogs, no accumulation was apparent during either the 7 or 28-day dosing periods. No obvious toxicities and no gross clinical or microscopic pathological effects were observed following these repeat exposures.

***Genotoxicity***

BLXA_4_-ME was not mutagenic in the in vitro bacterial reverse mutation (Ames) assay (4) and exhibited no clastogenic activity in an in vitro chromosome structural aberration assay in human blood lymphocytes.

***Oral irritancy studies***

Consistent with the investigation of a topical oral rinse product, an oral irritancy study was performed in rats to examine the effect of BLXA_4_-ME on the oral epithelium and on healing of oral epithelial abrasions. Prior to treatment with the BLXA_4_-ME oral rinse (15 male and 15 female rats) or control article (0.9% saline, 18 male and 18 female rats), the right buccal mucosa of each rat was abraded to induce minor oral irritation, while the left buccal mucosa was left intact. The 1μM BLXA_4_-ME oral rinse or saline was then applied to both sides of the mouth twice daily for up to 28 days. Macroscopic and microscopic observations were made during the dosing period and following a 14-day recovery period. The only findings were transient edema and erythema observed macroscopically in both the saline control and BLXA_4_-ME treated groups. No adverse microscopic effects of BLXA_4_-ME treatment were noted, and all macroscopic findings were mild and transient and had fully resolved within a few days after cessation of treatment. This study was intended to closely mimic the intended clinical route of administration, using an oral irritation model along with repeated daily topical dosing to achieve a maximal local effect. The observed effects were not unexpected for a surfactant-containing oral rinse product applied daily to abraded oral mucosa.

1. **Supplementary Figures and Tables**
   1. **Supplementary Figures**


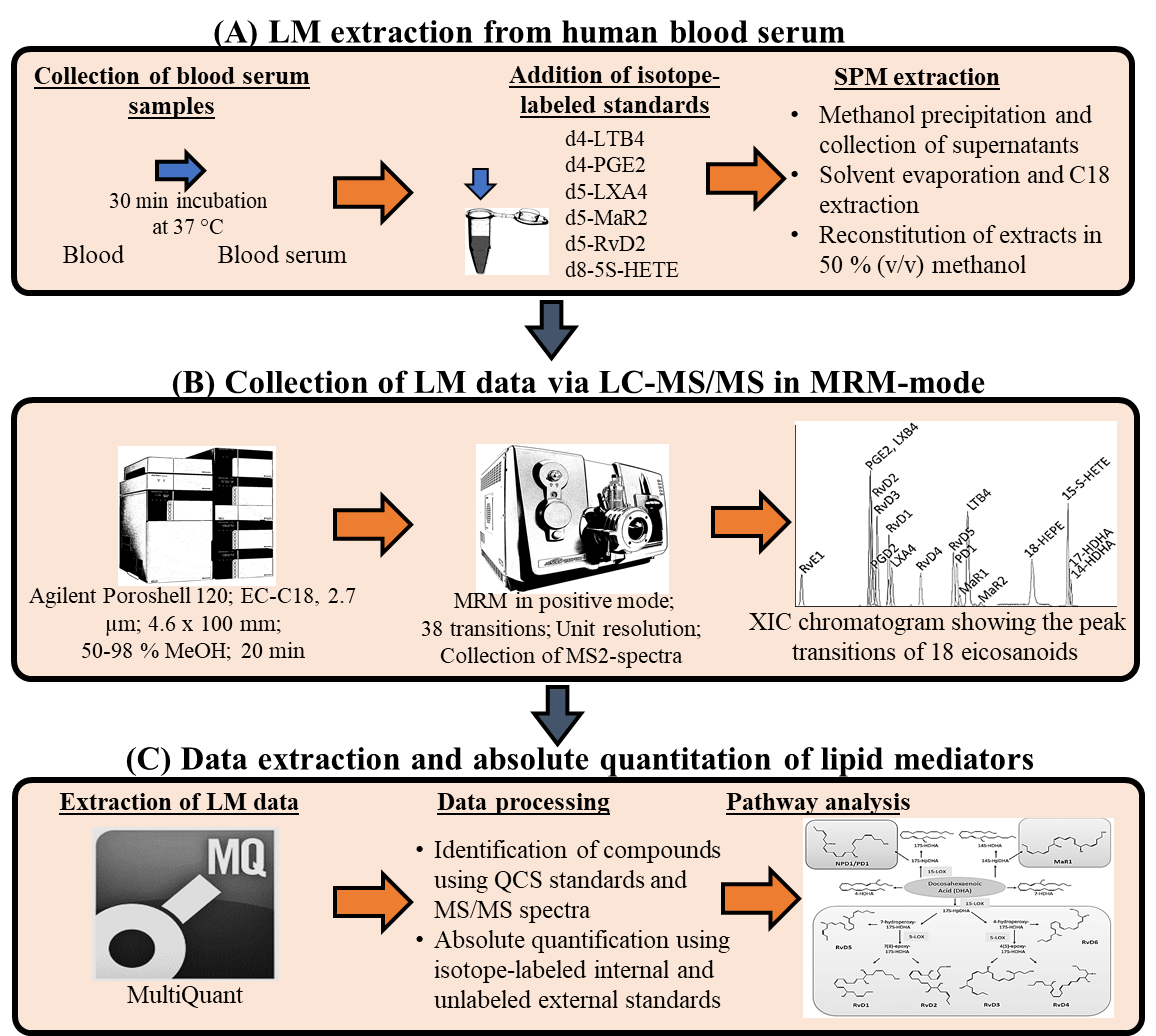


**Supplementary Figure 1.** **Targeted Lipid Mediator Lipidomics. (A)** Lipid mediator (LM) extraction from human blood serum. Serum samples were obtained from the peripheral blood collected at baseline and at 28 days post-baseline. After addition of isotope-labeled standards d4-LTB_4_, d4-PGE_2_, d5-LXA_4_, d5-MaR2, d5-RvD2, and d8-5S-HETE lipid mediators were extracted using C18-silica reverse-phase cartridges. **(B)** Collection of LM data via LC-MS/MS in multiple-reaction-monitoring (MRM) mode. The liquid chromatography coupled with tandem mass spectrometry system (QTrap 6500 equipped with a Shimadzu Nexera XL HPLC) was utilized for collection of LM data. An MRM-method was developed with signature ion fragments for each molecule to monitor and quantify the levels of targeted LMs. Calibration curves were obtained using both synthetic and authentic LM mixtures at 0.1, 1, 10, and 100pg. **(C)** Data extraction and absolute quantitation of lipid mediators. Quantification was carried out based on peak area of the MRM transition and the linear calibration curve for each compound.

- 1. **Supplementary Tables**

Supplementary Table 1. Inactive Components BLXA_4_ oral rinse

| **Component** | **Amount (%w/w)** |
| --- | --- |
| Saccharin sodium | 0.03 |
| Ethanol (95%) | 10.00 |
| Propylene glycol | 7.00 |
| Sodium lauryl sulfate | 0.25 |
| Sorbitol | 10.00 |
| Flavoring oil | 0.145 |
| Water | 72.575 |

BLXA_4_ = benzo-lipoxin A_4_; w = weight

Supplementary Table 2. Nonclinical safety pharmacology studies

| **Organ system evaluated** | **Species/**  **Strain** | **Method of administration** | **BLXA_4_‑ME Doses** | **Sex and number** | **Findings** |
| --- | --- | --- | --- | --- | --- |
| **In vitro** | | | | | |
| Cardiovascular (hERG channel) | hERG expressed in human embryonic kidney cells | In vitro | 1 μM  3 μM  10 μM | N/A | hERG current inhibition:  1.0 ± 0.5% at 1 µM  13.5 ± 1.2% at 3 µM  31.0 ± 0.6% at 10 µM  1.8 ± 0.6% in control  IC_50_ for the inhibitory effect of BLXA_4_-ME on hERG potassium current was estimated to be greater than 10 μM. |
| **In vivo** | | | | | |
| Central nervous system | Rat/Sprague Dawley | oral gavage | 200 μg/kg  500 μg/kg  2000 μg/kg | 5 males, 5 females per group | No apparent neuropharmacological effects |
| Respiratory | Rat/Sprague Dawley | oral gavage | 200 μg/kg  500 μg/kg  2000 μg/kg | 5 males, 5 females per group | No biologically relevant effects on respiratory rate, tidal volume, or minute volume |
| Cardiovascular | Dog/beagle | oral gavage | 200 μg/kg  500 μg/kg  2000 μg/kg | 4 males, Latin square design | No definitive changes in clinical observations, arterial pressure, heart rate, electrocardiogram, or body temperature |

hERG = human ether-à-go-go related gene

Supplementary Table 3. Mean pharmacokinetic parameters (BLXA_4_-FA) in Sprague Dawley rats following single 2000 μg/kg dose of BLXA_4_-ME by oral gavage

| Sex | C_max_  **(ng/mL)** | T_max_  **(hr)** | C_last_  **(ng/mL)** | T_last_  **(hr)** | AUC_(0-last)_  **(ng*hr/mL)** | AUC_(all)_  **(ng*hr/mL)** | AUC_(0–∞)_  **(ng*hr/mL)** | T_1/2_  **(hr)** |
| --- | --- | --- | --- | --- | --- | --- | --- | --- |
| Male  N = 15 | 1990 | 0.25 | 273 | 12 | 2966.8 | 4604.8 | 2967.4 | 1.06 |
|  | | | | | | | | |
| Female  N = 15 | 2070 | 0.25 | 18 | 8 | 3963.8 | 3999.8 | 3992.8 | 1.08 |

AUC = area under the curve; AUC_(0‑∞)_ = AUC from time zero to infinity; AUC_(0-last)_ = AUC from time zero to time of last quantifiable plasma concentration; AUC_(all)_ = AUC up to last time point; C_last_ = last quantifiable concentration; C_max_ = maximal plasma concentration; T_1/2_ = terminal plasma half-life; T_last_ = time of last quantifiable plasma concentration; T_max_ = time of maximum plasma concentration

**Supplementary** **Table 4. Mean pharmacokinetic parameters in beagle dogs following single 2000 μg/kg dose of BLXA_4_-ME by oral gavage**

| BLXA_4_-ME (N=4 per sex) | | | | | | | | |
| --- | --- | --- | --- | --- | --- | --- | --- | --- |
| **Sex** | **C_max_**  **(ng/mL)** | **T_max_**  **(hr)** | **C_last_**  **(ng/mL)** | **T_last_**  **(hr)** | **AUC_(0-last)_**  **(ng*hr/mL)** | **AUC_(all)_**  **(ng*hr/mL)** | **AUC_(0–∞)_**  **(ng*hr/mL)** | **T_1/2_**  **(hr)** |
| Male | 74.5 | 0.31 | 56.5 | 0.50 | 27.2 | 35.5 | 98.0^a^ | 0.487a |
| Female | 82.6 | 0.56 | 45.9 | 0.94 | 50.6 | 59.0 | 123.3^b^ | 0.600b |
| **BLXA_4_-FA (N=4 per sex)** | | | | | | | | |
| **Sex** | **C_max_**  **(ng/mL)** | **T_max_**  **(hr)** | **C_last_**  **(ng/mL)** | **T_last_**  **(hr)** | **AUC_(0-last)_**  **(ng*hr/mL)** | **AUC_(all)_**  **(ng*hr/mL)** | **AUC_(0–∞)_**  **(ng*hr/mL)** | **T_1/2_**  **(hr)** |
| Male | 532 | 0.25 | 13.0 | 4.5 | 433.1 | 442.9 | 458.4 | 1.52 |
| Female | 803 | 0.38 | 19.0 | 8.0 | 749.5 | 786.5 | 860.0 | 5.02 |

^a^N = 1

^b^N = 2

AUC = area under the curve; AUC_(0‑∞)_ = AUC from time zero to infinity; AUC_(0-last)_ = AUC from time zero to time of last quantifiable plasma concentration; AUC_(all)_ = AUC up to last time point; C_last_ = last quantifiable concentration; C_max_ = maximal plasma concentration; T_1/2_ = terminal plasma half-life; T_last_ = time of last quantifiable plasma concentration; T_max_ = time of maximum plasma concentration

1. **Supplementary Material References**

1. Sonis ST*, et al.* (1999) Validation of a new scoring system for the assessment of clinical trial research of oral mucositis induced by radiation or chemotherapy. Mucositis Study Group. *Cancer* 85(10):2103-2113.

2. Socransky SS*, et al.* (1994) "Checkerboard" DNA-DNA hybridization. *BioTechniques* 17(4):788-792.

3. Socransky SS*, et al.* (2004) Use of checkerboard DNA-DNA hybridization to study complex microbial ecosystems. *Oral microbiology and immunology* 19(6):352-362.

4. Ames BN, McCann J, & Yamasaki E (1975) Proceedings: carcinogens are mutagens: a simple test system. *Mutat Res* 33(1 Spec No):27-28.
